# Carotenoids in Cereal Food Crops: Composition and Retention throughout Grain Storage and Food Processing

**DOI:** 10.3390/plants8120551

**Published:** 2019-11-28

**Authors:** Daniela Trono

**Affiliations:** Consiglio per la Ricerca in Agricoltura e l’Analisi dell’Economia Agraria, Centro di ricerca Cerealicoltura e Colture Industriali, S.S. 673, Km 25,200, 71122 Foggia, Italy; daniela.trono@crea.gov.it

**Keywords:** carotenoids, cereal grains, biofortification, storage, processing

## Abstract

Carotenoids are C40 isoprenoids synthesized by plants, as well as some bacteria, fungi and algae, that have been reported to be responsible for a number of benefits conferred on human health. The inability of animals and humans to synthesize de novo these compounds is the reason why they must be introduced from dietary sources. In cereal grains, carotenoids are important phytochemicals responsible for the characteristic yellow colour of the endosperm, which confers nutritional and aesthetic quality to cereal-based products. Cereals are staple foods for a large portion of the world population, and the biofortification of cereal grains with carotenoids may represent a simple way to prevent many human diseases and disorders. Unfortunately, evidence exists that the storage and processing of cereal grains into food products may negatively impact their carotenoid content; so, this loss should be taken into consideration when analysing the potential health benefits of the cereal-based products. Focusing on the recent updates, this review summarizes the chemical composition of the carotenoids in the grains of staple cereals, including wheat, maize, rice and sorghum, the main factors that affect their carotenoid content during storage and processing and the most fruitful strategies used improve the grain carotenoid content and limit the carotenoid post-harvest losses.

## 1. Introduction

Carotenoids are C40 isoprenoids synthesized by plants, as well as some bacteria, fungi, and algae, comprising a large family with more than 700 yellow, orange or red fat-soluble pigments that include carotenes (non-oxygenated molecules) and xanthophylls (oxygenated molecules). The inability of animals and humans to synthesize carotenoids de novo is the main reason why they rely upon diet for these essential compounds. Carotenoids have been reported to be responsible for a number of benefits conferred on human health. In particular, α-carotene, β-carotene and β-cryptoxanthin are important for their provitamin A activity. After ingestion they are converted into retinol (i.e., vitamin A), which serves as the precursor of the light sensor molecules in the retina. An adequate intake of provitamin A carotenoids can prevent degenerative eye damages, such as night blindness, xerophthalmia, Bitot’s spot, corneal ulcerations and lesions [1]. In addition, carotenoids act as antioxidants and their intake has been linked with reduced risk of a number of chronic diseases. Xanthophylls, such as lutein and zeaxanthin, are the major components of macular pigments; they react as antioxidants against free radicals and reactive oxygen species, thus protecting the retina against peroxidation and photo-damage and impeding the onset of age-related macular degeneration [2], which is the leading cause of blindness in the Western world [3]. Lycopene improves the endothelial function and reduces the incidence of coronary heart disease [4], whereas β-carotene, lycopene and retinol have been found to prevent Alzheimer’s disease symptoms [5]. An association has also been reported between the intake of foods rich in carotenoids and reduced risk of type 2 diabetes [6], colorectal cancer [7] and obesity [8]. 

Vitamin A deficiency is widespread throughout people resident in under-developed and developing countries, particularly in Central and West Africa and South-Central Asia, where it is responsible for a million or more instances of death and blindness each year among preschool-age children and pregnant women [9]. In these areas, diet is mainly based on the consumption of a single staple cereal crop; this implies a lack of dietary diversity that in combination with the small amounts of carotenoids (particularly of provitamin A carotenoids) in cereal grains, is a risk factor for vitamin A deficiency. Similarly, chronic diseases (e.g., cardiovascular diseases, diabetes, obesity and cancers) are the largest cause of death in the industrialized countries and the ingestion of carotenoids through the diet may have protective effects against such chronic diseases and contribute to a healthy life and well-being [10]. So, the development of biofortified cereal crops with high carotenoid content represents a powerful means to help alleviate vitamin A deficiency in the poor regions of the world, as well as other nutritional and health problems worldwide. 

Unfortunately, carotenoids are very sensitive regarding exposure to heat, oxygen, light, acids and transition metals, as well as oxidative enzymes, and evidence exists that the storage and processing of grains into food products may negatively impact on their carotenoid content; so, this loss should be taken into consideration when analysing the potential health benefits of the cereal-based products. Globally, cereal grains are harvested during a short period of time but consumed throughout the year. So, buffer stocks of cereal grains and flours are maintained for extended periods of time in order to supply domestic and industrial needs. The low water content in cereal grains and flours favours their conservation over time. However, during long-term storage, high temperatures, humidity and oxygen in the storage space, as well as excessive moisture in the stored products, may have an important impact on the content and/or the composition of carotenoid pigments. Moreover, cereal grains need to be processed before consumption. Both industrial processing and domestic preparation methods (e.g., soaking, boiling, baking, toasting, frying, extrusion, fermentation and nixtamalization) expose foods to high temperatures and lead to the destruction of the food cellular structure and to the increase of the surface area, all phenomena that may have detrimental effects on carotenoid compounds. The major alterations undergone by carotenoids during storage and processing are isomerisation from *trans*- to *cis*-isomers and oxidation by different oxidizing agents; carotenoid loss due to oxidation appears to be the dominant mechanism in foods [11].

## 2. Carotenoid Metabolism

Carotenoid content, composition and distribution across the kernel differ significantly among cereals. Maize presents higher carotenoid content compared to non-corn cereals, with zeaxanthin as the dominant carotenoid (Table 1). Among non-cereals, lutein is the main component in wheat [12], whereas β-carotene and zeaxanthin are the predominant carotenoids in sorghum followed by lutein [13]; very low levels of carotenoids, mainly β-carotene and lutein, are present in raw rice [14] (Table 1). Carotenoids are unevenly distributed across the cereal kernel. In maize, most of the contribution to the total carotenoid content (>92–106%) comes from the endosperm, whereas in non-corn cereals the contribution to total carotenoid content ranges from 20.5–70.6% in the endosperm, 3–10% in the germ and 20–72% in the bran [15]; in particular, in raw rice, the carotenoid compounds are present only in the bran and in the outer endosperm, whereas they are completely absent in the middle and core endosperm [14].

### 2.1. Biosynthesis

**Figure 1:**
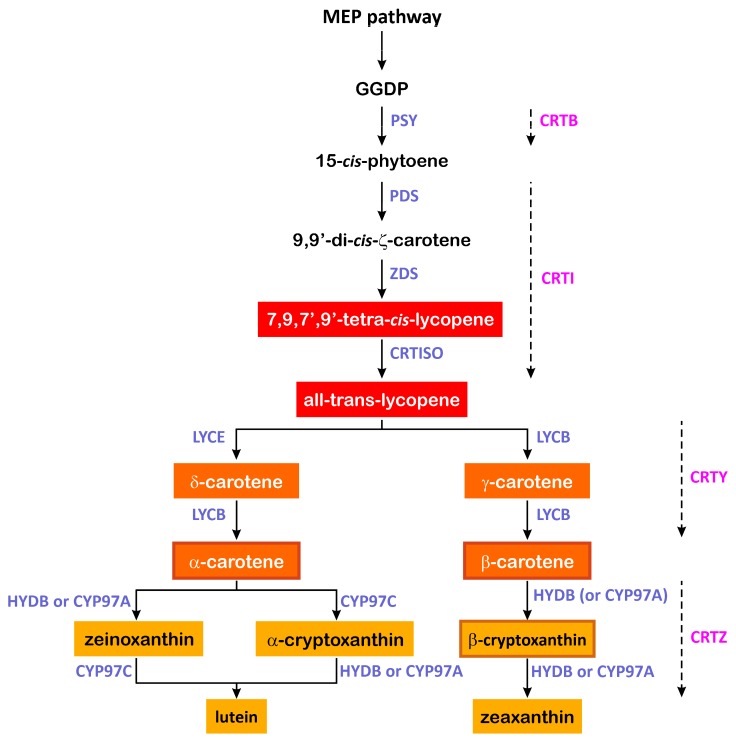
Carotenoid biosynthesis pathway in higher plants and the corresponding steps in bacteria. MEP, methylerythritol 4-phosphate; GGPP, geranylgeranylpyrophosphate; PSY, phytoene synthase; PDS, phytoene desaturase; ZDS, ζ-carotene desaturase; CRTISO carotenoid isomerase; LYCB, lycopene β-cyclase; LYCE, lycopene ε-cyclase; HYDB (also known as BCH), β-carotene hydroxylase; CYP97A and CYP97C, heme-containing cytochrome P450 carotene β- and ε-ring hydroxylases; CRTB, bacterial phytoene synthase; CRTI, bacterial phytoene desaturase/isomerase; CRTY, bacterial lycopene β-cyclase; CRTZ, bacterial β-carotene hydroxylase. Provitamin A carotenoids are boxed.

The carotenoid biosynthesis starts with the condensation of geranylgeranylpyrophosphate (GGPP), a precursor from the upstream methylerythritol 4-phosphate (MEP) pathway (Figure 1). The availability of the precursor derived from the MEP pathway represents a bottleneck that influences the flux through the entire pathway [16]. Indeed, the expression levels of the 1-deoxyxylulose-5-phosphate synthase (*DXS*), 1-deoxy-D-xylulose5-phosphate reductoisomerase (*DXR*) and 1-hydroxy-2-methyl- 2-(*E*)-butenyl 4-diphosphate reductase (*HDR*) genes involved in the MEP pathway were found to be positively correlated to the carotenoid content in maize endosperm [17]. The condensation of the GGPP produces the C40 15-*cis*-phytoene, which is the first compound in the carotenoid biosynthetic pathway (Figure 1). This reaction is catalysed by phytoene synthase (PSY in plants, CRTB in bacteria) and represents the first rate-limiting step for the endosperm carotenoid biosynthesis [18]. Phytoene undergoes four desaturations catalysed by the phytoene desaturase (PDS) and ζ-carotene desaturase (ZDS) to form lycopene, whereas carotenoid isomerase (CRTISO) is responsible for the conversion of the poly *cis*-compounds to their all-*trans* forms. In bacteria, the desaturation and isomerisation reactions are catalysed by only one enzyme, the bacterial phytoene desaturase/isomerase (CRTI) (Figure 1). Lycopene is then cyclized by lycopene ε-cyclase (LCYE) and/or lycopene β-cyclase (LCYB) (CRTY in bacteria) that incorporate ε- and β-rings, respectively, and produce α- and β-carotene (Figure 1). Carotenoid hydroxylase enzymes specific to the β-ring (HYDB or CYP97Ain plants, CRTZ in bacteria) and the ε-ring (CYP97C) catalyse the double hydroxylation of α-carotene and β-carotene and lead to the formation of the xanthophylls lutein and zeaxanthin, respectively (Figure 1). The cyclization of lycopene plays an important role in modulating the β-β/β-ε branch ratio. Indeed, the overexpression of the *LCYB* gene shifts the pathway toward the β-β branch, whereas overexpression of the *LCYE* gene has the opposite effect [19].

**Figure 2:**
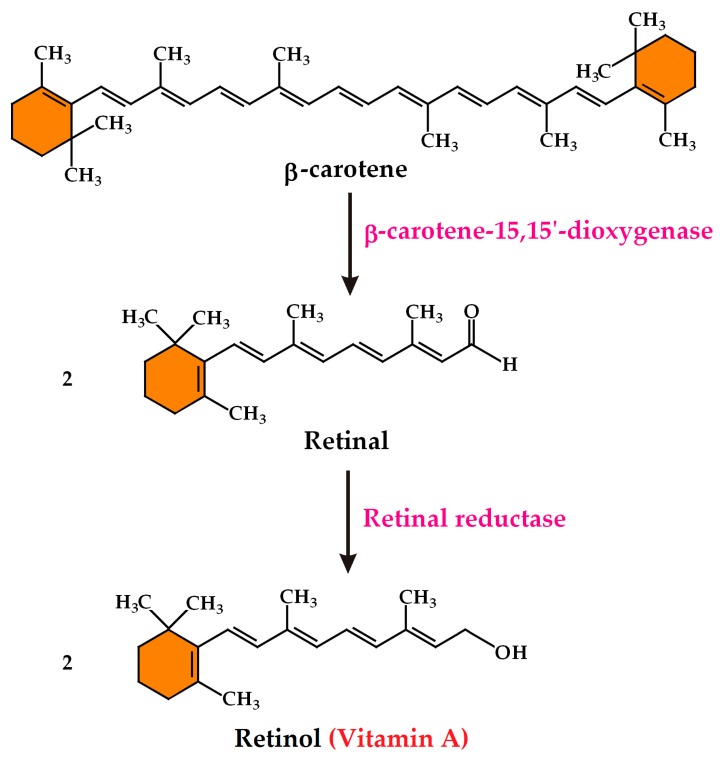
Conversion of β-carotene to retinol (vitamin A). β-Carotene-15,15-dioxygenase catalyses the central cleavage of β-carotene that yields two molecules of retinal. Subsequently, retinal reductase catalyses the reduction of retinal to retinol.

Those carotenoids that possess a β-ring have provitamin A activity and can be converted into vitamin A (retinol) by the human body. β-Carotene with two β-rings has the highest provitamin A activity as one molecule of β-carotene gives two molecules of retinol (Figure 2), whereas α-carotene or β-cryptoxanthin, with only one β-ring, have half the provitamin A activity compared to β-carotene [20]. In the carotenoid biosynthetic pathway, the reactions catalysed by HYDB, which convert the provitamin A carotenes into the non-provitamin A xanthophylls, are responsible for the depletion of the provitamin A activity. 

### 2.2. Degradation

Degradation of carotenoid pigments occurs via specific and non-specific mechanisms. The specific mechanisms involve the carotenoid dioxygenases, a family of enzymes that catalyse the degradation of carotenoids to apocarotenoids. This family includes nine-*cis*-epoxycarotenoid dioxygenases (NCEDs) that are involved in the synthesis of the abscisic acid, and carotenoid cleavage dioxygenases (CCDs) that give rise to strigolactones and volatile compounds responsible for the aroma released by vegetables [21]. Non-specific mechanisms include enzymatic and non-enzymatic oxidation. The enzymatic oxidation is triggered by lipoxygenases (LOXs). LOXs are non-heme iron-containing dioxygenases that catalyse the hydroperoxidation of polyunsaturated fatty acids containing *cis,cis*-1,4-pentadiene structures (e.g.,linoleic and linolenic acids); the radicals produced during the intermediate steps of substrate hydroperoxidation can cause the oxidation of the carotenoid pigments [22].

The non-enzymatic carotenoid destruction is due the characteristic conjugate double-bond structure found in carotenoids, which represents an electron-rich system susceptible to reactions with electrophilic oxidizing agents. Oxidation leads to the generation of epoxy- and peroxyl-derivatives of carotenoids, which decompose into apocarotenoids and finally form a number of products which are in part identical to those formed enzymatically [23].

## 3. Wheat

### 3.1. Composition

Wheat is the most important staple crop in the temperate regions where it represents a primary source of energy (starch), as well as an important source of proteins, fibre and beneficial phytochemicals. Moreover, there is an increasing demand for wheat in countries undergoing urbanization and industrialization. The increasing demand is linked to the unique properties of the gluten protein fraction of wheat that allows the processing into unique food products [24]. The most important modern wheat species grown throughout the world are hexaploid bread wheat and tetraploid durum wheat, which differ from one another for their genome, grain composition and derived end-products. Although carotenoids are generally minor components in wheat grains, the high amount of wheat-based foods consumed in most human cultures might make wheat an important carotenoid source for humans and may help to prevent both chronic diseases and deficiencies.

Since consumers usually prefer white bread made from bread wheat, white flour varieties with low carotenoid content in the grains have been usually selected in bread wheat. On the contrary, the high carotenoid content is an important characteristic in the utilisation of durum wheat for pasta production and for this reason the improvement of carotenoid grain content has always been one of the main targets in the traditional breeding of durum wheat [25]. Consistently, Digesù and coworkers [26] found that modern durum wheat varieties show significantly higher values of total carotenoid content compared to old varieties. In particular, the total carotenoid content was found to be 1.178–3.345 µg/g, 1.352–3.532 µg/g and 1.857–4.416 µg/g in the varieties released before 1971, between 1971 and 1990 and between 1991 and 2008, respectively; overall, lutein was the main component of carotenoids (0.721–3.078 µg/g), followed by zeaxanthin (0.088–0.184 µg/g) and β-carotene (0.007–0.033 µg/g), whereas α-carotene and β-cryptoxanthin were minor components [26] (Table 1). Lutein was the major carotenoid also in bread wheat but its concentration in this species was significantly lower compared to durum wheat (0.391–1.890 µg/g) [27,28,29,30] (Table 1). *Hordeum chilense*, a diploid wild barley with a high potential as a genetic donor to increase the carotenoid content in wheat grains, was used for the development of bread wheat–*H. chilense* translocation lines for chromosome 7H^ch^, where the *PSY* gene is located [30]. An increase in the total carotenoid content was observed in the translocation lines that reached values two-fold higher compared to the control bread wheat (1.133–1.215 µg/g vs. 0.603 µg/g), mainly ascribable to the higher accumulation of free lutein (0.897–1.020 µg/g vs. 0.391 µg/g) [30] (Table 1). All together, these results demonstrate marked variations in the carotenoid composition and concentration of wheat grains. Such information is useful for plant breeders in screening and selecting cultivars with high yellow colour and enhanced phytochemical concentration.

Mapping studies carried out both in bread and durum wheat identified major quantitative trait loci (QTLs) on group 7 chromosomes that accounted for up to 60% of the observed variation in the carotenoid content in wheat grains. The *PSY1* gene was found to co-segregate with major QTLs on the chromosomes 7A and 7B of bread and durum wheat [31,32,33] and was proposed as candidate gene for high carotenoid content in wheat grains. So, functional markers were developed on both chromosome A and B *PSY1* genes and some of them were also validated [33,34,35]. In particular, Patil and coworkers [33] developed a marker on the chromosome A *PSY1* gene and used it in marker-assisted selection (MAS) programs to improve the carotenoid content in two élite varieties of Indian durum wheat; the new lines obtained showed two-fold increase in total carotenoid content compared to the corresponding parental genotypes (5.00–7.70 µg/g vs. 3.26–3.57 µg/g) (Table 1).

In addition to these major QTLs at *PSY1* loci, QTLs with smaller phenotypic effects have also been detected [32,36]. Among these, a new QTL for high carotenoid content was detected on chromosome 2A of bread wheat, which co-segregated with the *ZDS* gene and accounted for the 11% of the variation in grain carotenoid content; a functional marker was designed based on the polymorphisms of two alleles at this locus and, once validated, will certainly be useful in future programs of marker-assisted breeding [36].

A transgenic approach has also been used by Cong and coworkers [37]. The authors generated transgenic bread wheat lines overexpressing the maize gene encoding the PSY (*ZmPSY*) together with the *CRTI* gene from *Pantoea ananatis* (*PaCRTI*). Compared with the nontransgenic genotype the transgenic lines presented up to a 10.8-fold increase in total carotenoids that reached values as high as 4.96 μg/g (Table 1). By simultaneously introducing the *PaCRTB* and the *PaCRTI* genes into a low carotenoid bread wheat varieties, the same authors achieved 8-fold increase in the total carotenoid content up to 4.76 μg/g and 76-fold increase in the provitamin A carotenoid content that reached 3.82 μg/g, most of which was represented by β-carotene (3.21 μg/g) [38] (Table 1). Further increase in the β-carotene content was obtained by the same research group by combining the endosperm-specific silencing of the *HYD* gene and the overexpression of the bacterial *CRTB* gene [39]. Through this combination of methods, significant levels of β-carotene accumulation were obtained, corresponding to an increase of up to 31-fold and a concentration of up to 5.04 μg/g (Table 1).

### 3.2. Storage

Wheat for human consumption can be stored as a grain but, as it is traditionally processed before consumption, it is more frequently stored as flour for more or less long periods of time. The effect of short-term storage (90 days) on the carotenoid content of durum wheat grains was assessed at different storage temperatures (4 °C, 20 °C and 37 °C) by Mellado-Ortega and coworkers [40]. The authors observed that at the lower storage temperature an increase in the carotenoid content after the first 30 days of storage was observed that ranged between 31% and 65%; the authors hypothesized that this phenomenon was linked to the dormancy breakage that generally occurs in grains stored at this temperature. No significant losses were observed after 90 days of storage at 20 °C, whereas an average decrease of 30% was observed at 37 °C [40]. Carotenoid losses in durum wheat grains were even higher after longer periods of storage. Interestingly, xanthophyll esters appeared as a consequence of storage and significant differences were observed between free and esterified pigments, with smaller losses observed for the latter. In particular, free carotenoids completely disappeared after 12 months of storage at 50 °C, whereas esterified pigments under the same conditions of storage declined by 76%. Lower reduction in the esterified compared to free pigments was also observed at 37 °C (28% vs. 62–79%) [41]. The in vivo esterification of xanthophylls is known to be responsible for the abundance and stability of carotenoids in different fruits and vegetables [42], but in cereal grains this process seems to be absent or at very low levels [40]. However, under ex vivo conditions, xanthophylls in grains are susceptible to esterification probably mediated by lipases, which are concentrated in the bran and, under low-water conditions, may catalyse esterification or transesterification reactions ([43] and references therein). The higher retention of the esterified compared to free grain xanthophylls confirms the role of esterification as a mechanism that provides extended stability to these pigments.

Compared to grains higher carotenoid losses were observed after short-term storage (90 days) of durum wheat whole-grain flour, with an average reduction of 50% at 4 °C and 20 °C, 78% at 37 °C and 94% at 50 °C [59]. Similar degradation values were also reported for bread wheat whole-grain flour (4% at −20 °C, 23% at 5 °C, 35% at 20 °C, 61% at 30 °C and 88% and 38 °C) and white flour (8% at 20 °C, 24% at 5 °C, 49% at 20 °C, 62% at 30 °C and 91 at 38 °C) [60]. The higher losses observed in the flours compared to the grains suggest that the flour is more vulnerable than grains with respect to the storage conditions, and this is probably due to the loss of cell integrity caused by the milling process. Farrington and coworkers [61] also evaluated the effect of very long-term storage (5 years at 12 ± 2 °C) on the carotenoid content of weak, medium and strong flour from bread wheat and observed a carotenoid loss of 70%, 67% and 48%, respectively. Interestingly, as already observed in the grains, also in the flour esterified xanthophylls appeared with time and showed greater stability than the corresponding free forms. For instance, 93% reduction was observed for free lutein in durum wheat whole-grain flour after 90 days of storage at 50 °C, whereas, under the same conditions, the esterified lutein decreased only by 50% [59]. This information will be useful for the optimization of storage conditions of wheat grains and flours and could also be used in crop biofortification programs for the selection of cereal varieties with an enhanced content of esterified xanthophylls.

### 3.3. Processing

Wheat flours are undoubtedly the products derived from wheat with the widest applications. Flour obtained from bread wheat is generally used for the production of leavened bread and other baked goods, whereas semolina obtained from durum wheat is primarily associated with the production of pasta, couscous and burghul.

During the milling process, a loss of carotenoid pigments was observed in durum wheat semolina of about 8% [62]. In addition, evidence has been reported that high amounts of ash (deriving from the bran layers) in the semolina increase the levels of oxidative enzymes, which in turn increase the carotenoid degradation and loss [63]. So, partly sacrificing semolina yield can help to reduce carotenoid losses associated to the milling process.

During bread making, a limited carotenoid degradation was observed by Hidalgo and coworkers [64] after dough kneading and leavening (15% and 3%, respectively), whereas baking determined a significant carotenoid loss in bread crust (29%) but not in bread crumb (3%). Conversely, Leenhardt and coworkers [65] reported that major carotenoids losses (66%) occurred during kneading, whereas lower losses were observed after fermentation (10%) and baking (36%). Similar values were also reported for coloured-grain wheat flours [66], with a loss in the total carotenoid content of 62% and 27% after dough preparation and baking, respectively. As already observed under storage, lutein esters were more stable compared to free lutein also under dough preparation. Interestingly, bread wheat dough obtained with some lactic acid bacteria presented a significant increase (from 60% to 100%) in the carotenoid content probably due to an increased mobilization of these membrane-associated lipophilic compounds from the cereal matrix [67].

As far as pasta processing, relevant carotenoid losses were observed during longer kneading-extrusion phase (48%), whereas the drying step did not cause significant variation in the carotenoid content [64]. Ficco and coworkers [68] found that the effect of processing differed in pasta produced from semi-wholemeal compared to that obtained from semolina. In particular, the authors observed that the extrusion determined a decrease in carotenoid content that was higher in pasta obtained from the semi-wholemeal compared to pasta obtained from semolina (32% vs. 16%); conversely, after cooking carotenoid content was found to decrease in pasta obtained from semolina (14%) and to increase in pasta obtained from semi-wholemeal (34%). Evidence has been reported that carotenoid losses during pasta processing are mainly due to LOX activity. Indeed, Trono and coworkers [69] observed that carotenoid losses during pasta processing ranged from 28% to 34% in durum wheat varieties characterized by high LOX activity in semolina and from 0% to 6% in varieties with low LOX activity in semolina. The LOX1-encoding gene family mapped on chromosome 4B (*Lpx-B1*) was found to account for most of the total LOX activity in durum wheat grains and a deleted allelic variant (*Lpx-B1.1c*) was identified that was found to be associated with very low LOX activity in semolina [70]. This allele is currently being used as a molecular marker in assisted breeding programs for the selection of new durum wheat lines characterized by low LOX activity in semolina and, consequently, by high carotenoid retention during pasta processing [71,72].

## 4. Maize

### 4.1. Composition

Maize is the third most important cereal staple food crop worldwide after wheat and rice and is a major cereal staple food for African consumers. So, fortification of maize would have a crucial role in alleviating vitamin A deficiency in developing countries and reducing the risk of chronic disease in industrialized countries. Maize is the only cereal crop that contains considerable amounts of carotenoid pigments with a wide natural variation across genotypes [73]. A panel of 201 maize inbreds with kernel colour ranging from light yellow to dark orange revealed a total carotenoid content that ranged from 9.55 to 62.96 μg/g [44] (Table 1). The most abundant carotenoid compounds in maize grains were zeaxanthin (1.44–32.40 μg/g) and lutein (1.23–23.93 μg/g), but provitamin A carotenoids, β-carotene, α-carotene and β-cryptoxanthin were also present at levels significantly higher (0.31–3.27 μg/g, 0.45–2.65 μg/g and 0.13–5.17 μg/g, respectively) compared to other non-corn cereals [44,45] (Table 1). 

Due to the considerable natural genetic variability for carotenoid composition, conventional breeding has been efficiently applied to improve the carotenoid content in maize grain. Most of the initial efforts have been focused on maize from temperate regions that reached levels of provitamin A carotenoids in the endosperm as high as 19 μg/g [74]. These improved lines were then used in conventional breeding crosses to develop varieties adapted to sub-Saharan Africa’s areas; the first provitamin A-fortified maize variety was released in Zambia in 2012 and contained provitamin A carotenoids at levels of 7–8 μg/g [75], which corresponded to half of the target level of 15 µg/g set for the fortification of provitamin A maize by HarvestPlus, a global partnership programme that aims to the development and promotion of biofortified crops for a better nutrition [74]. 

Association mapping studies were subsequently carried out that revealed the existence of favourable polymorphisms in the *LYCE* and the β-carotene hydroxylase 1 (*CrtRB1)* genes; so, PCR-based markers were designed for both *LCYE*- and *CrtRB1*-based polymorphisms and used in MAS programs to develop fortified maize lines with β-carotene concentrations up to 26 μg/g (Table 1) and total provitamin A as high as 30 µg/g [46]. These improved sources of provitamin A were used to develop new tropical hybrids with a provitamin A content of 9–11 µg/g that were released in Zambia in 2015 [75]. Breeding programs are still in course to develop tropical hybrids that meet or exceed the full 15 µg/g provitamin A HarvestPlus target. The favourable polymorphisms in the *CrtRB1* gene were also introgressed from CYMMIT maize lines into Indian elite inbreds that were used as parents for high yielding commercial maize hybrids in India [47]. The β-carotene concentration in the improved inbred lines ranged from 8.6 to 17.5 μg/g and the hybrids developed from these improved parental inbreds showed further enhanced β-carotene content up to 21.7 μg/g [47]. 

Transgenic approaches to improve the provitamin A carotenoid content in maize endosperm have also been attempted. The overexpression of the *PaCRTB* and *PaCRTI* genes determined an increase in the total carotenoid content up to 34-fold, with a preferential accumulation of β-carotene up to 8.64 µg/g [48] (Table 1). Silencing by RNA interference of the *BCH2* gene and the introgression of the RNAi-*ZmBCH2* silencing cassette into high carotenoid lines NC356 (~74 µg/g) and PSY (~54 µg/g) resulted in a significant increase of the β-carotene content in the corresponding hybrids that reached values of 21–30 µg/g [49] (Table 1). Plants with extraordinary levels of β-carotene were obtained in multiplex-transgenic plants by Zhu and coworkers [50] through combinatorial nuclear transformation. In particular, the combined expression of *ZmPSY1* and *PaCRTI* genes generated an orange–red phenotype that accumulated up to 57.35 µg/g β-carotene.

### 4.2. Storage

Maize for human consumption is stored as whole grain or as flour. So, carotenoid degradation during grains and flour storage under different conditions has been widely investigated. Burt and coworkers [76] assessed the variations in carotenoid content in high-carotenoid (~60 µg/g) maize lines after exposure to different drying treatments and storage conditions: freeze-drying and storage at −80 °C; room temperature drying and storage; 90 °C drying and room temperature storage. They observed an immediate carotenoid loss (15–45%) after drying at both room temperature and 90 °C but not under freeze-drying. Carotenoid levels in freeze-dried samples remained unchanged also after four months of storage at −80 °C, whereas the other two drying treatments stored in the dark at room temperature presented further carotenoid losses (24–61%) [76].

The effect of different storage temperatures (4 °C, 22.5 °C and 55 °C) and humidity (57% and 75%) was also evaluated on maize genotypes biofortified with provitamin A (up to 16.1 µg/g) in the HarvestPlus breeding programs [77]. The rate of carotenoid degradation in maize grains stored at 4 °C and 22.5 °C was significantly lower than that observed at 55 °C. Moreover, at the highest temperature the provitamin A content zeroed after 27 months of storage, whereas grains stored at 4 °C and 22.5 °C maintained 20–40% of their initial provitamin A content until the end of the period examined (53 months of storage). Similarly, low humidity (11%) led to a carotenoid degradation rate that was significantly lower than that observed at high humidity values (57% and 75%). Interestingly, differences were also observed between the full flint-and the dent-type kernels, with the latter that showed a β-carotene degradation rate under storage two-fold higher compared to the former [77]. The flint-type kernel is more vitreous than dent-type kernel and a higher concentration of carotenoids is typically located in the vitreous compared to the soft starch endosperm [78]; moreover, the higher hardness and density of the dent-type kernel may protect carotenoid from degradation and reduce the rate of carotenoid loss. Overall, these observations confirm the role of temperature and humidity in the maintenance of carotenoid stability during the storage and highlight that a genotype effect also exists that may potentially allow the development of inbred lines with improved stability during grain storage.

Carotenoid retention was found to be also affected by the type of storage container. After 180 days of storage, a complete retention of provitamin A carotenoids was observed in orange maize grains stored in aluminium bags with oxygen absorbers, followed by Purdue Improved Crop Storage (PICS) bags and silo with candle with a 57% retention, whereas grains in woven bags, silo without candles and ears in woven bags had the lowest carotenoid retention (between 51% and 48%) [79].

As far as the storage of maize flour, evidence has been reported that retention of provitamin A carotenoids depends on flour type. After 4 months of storage, coarser hammer meal presented higher retention (73–105%) than finer refined meal (64–90%) and, as already observed for the grains, the retention in the aluminium bags was the highest among all the storage containers [79]. β-Cryptoxanthin was more stable than β-carotene both in grains and flour under all the storage and the packaging conditions. Consistently, when the retention of carotenoids was assessed in high-xanthophyll and high-β-carotene maize genotypes after long-term storage at different temperatures (−20 °C, 22 °C and 37 °C), greater losses of carotenoids were observed at all the conditions of storage in high-β-carotene maize compared with high-xanthophyll maize [80]. In light of this, it is feasible that maize grains fortified with higher proportions of β-cryptoxanthin compared to β-carotene could have higher impact on the alleviation of vitamin A deficiency.

### 4.3. Processing

Maize is consumed as whole-grain and processed products. Whole-grain maize is consumed directly cooked on the ear, canned or as popcorn, whereas milled maize can be processed into homemade dishes such as porridge, polenta and other traditional preparation methods (that often include further processing, such as fermentation, soaking and nixtamalization), or into other industrial products that include flaked products, fried chips and other snack foods. 

The effect of cooking on the carotenoid content of maize grains and processed foods differed depending on the cooking method. Domestic cooking of maize ears by steaming and boiling determined a significant increase in total carotenoid content (from 41% to 180%), as well as in lutein (from 36% to 232%), zeaxanthin (from 48% to 457%), β-cryptoxanthin (from 22% to 405%) and β-carotene (from 16% to 88%) content [81]. This is probably due to the release of the bound carotenoids from the matrix food [82]. Conversely, baking was found to decrease the total carotenoid content by almost 70% [83]. As far as the effect of commercial canning, no significant differences were observed between total carotenoids in canned and fresh grains [84].

Carotenoid retention in high-β-cryptoxanthin maize was determined in muffins, non-nixtamalized tortillas, porridge and fried puffs made from whole-grain and sifted flour. Boiling whole-grain flour into porridge resulted in the highest retention of all cooking and sifting methods (112%), whereas deep-frying had the lowest carotenoid retention (67–78%) [80]. Similar findings were reported by other studies on provitamin A biofortified maize; β-carotene retention of 76% and 75% was observed for African traditional fermented and unfermented porridge, respectively [85], whereas nixtamalization and frying during the preparation of Mexican-inspired products led to a retention of 64% [86]. 

Cueto and coworkers [87] compared the lutein and zeaxanthin content in cornflakes prepared by the traditional process (maize grits cooking, drying, flaking and toasting) and by extrusion. After the cooking stage of the traditional process 60% and 40% reduction in the content of lutein and zeaxanthin, respectively, was observed. For both compounds the reduction reached 80% after toasting. Conversely, extruded maize showed only 35% reduction in lutein and zeaxanthin content. A similar loss was also observed for provitamin A carotenoids by Ortiz and coworkers [88] under extrusion at 25% moisture, whereas extrusion at 35% moisture resulted in even lower loss (7–30%). These findings suggest the extrusion processing is crucial for preserving biofortified maize end-products. 

## 5. Rice

### 5.1. Composition

Rice is an important staple food in developing countries, but it contains the lowest content of carotenoid pigments among all the cereals the major brown rice carotenoids are β-carotene and lutein (up to 0.150 µg/g and 0.109 µg/g, respectively), whereas zeaxanthin levels are lower (up to 0.037 µg/g) [14] (Table 1). These carotenoids accumulate almost exclusively in the bran and in the outer endosperm, whereas they are absent in the core endosperm [14]. So, the consumption of polished rice-based foods in a non-diverse diet leads to vitamin A deficiency. Due to the absence of the rice germplasm of genotypes able of accumulating carotenoids in the core endosperm, genetic engineering rather than conventional breeding was the only way to induce the accumulation of carotenoids in rice grains. The genetically modified rice obtained is known as ‘Golden Rice’, a name that is derived from the yellow colour of the grains. The research that led to the development of ‘Golden Rice’ was initiated by Ye and coworkers [51]. As the biosynthesis of carotenoids in rice endosperm is blocked at the first enzymatic step catalysed by the PSY and a limited flux is also present in the subsequent desaturation reaction, the authors transformed the Japonica rice cultivar Taipei 309 with the *Narcissus pseudonarcissus PSY* (*NpPSY*) gene together with the *PaCRTI* gene; they also co-transformed with the *Narcissus pseudonarcissus LYCB* (*NpLCY*) gene to lead the pathway towards the β-carotene biosynthesis. The mutant lines obtained, which are known as the prototype of ‘Golden Rice’, contained up to 1.6 µg/g carotenoids (Table 1) and produced β-carotene (up to 0.7 µg/g) regardless of the presence or absence of the *NpLCY* gene [51,52,53]. In the subsequent years, scientists produced several transformation lines from Japonica, Javanica and Indica rice cultivars characterized by increasingly high levels of carotenoids by using a modified vector in which the *NpPSY* gene was under the control of an endosperm-specific promoter [89,90], whereas in the experiments of Ye and coworkers [51] this gene was under the control the CaMV 35S promoter. In particular, Syngenta scientists transformed the Javanica cultivar Cocodrie and obtained a new version of ‘Golden Rice’, known as ‘Golden Rice-1′, that contained as much as 6.0 µg/g carotenoids in the endosperm [54] (Table 1). In 2005, Syngenta scientists substituted the *NpPSY* gene with the *ZmPSY* gene and this led to the development of a new version of ‘Golden Rice’, the ‘Golden Rice-2′ that was characterized by amounts of carotenoids in the endosperm up to 36.7µg/g, of which 84% was β-carotene (up to 30.9 µg/g) [55] (Table 1).

Recently, Bai and coworkers [56] obtained transgenic lines by expressing the *ZmPSY* and the *PaCRTI* genes alone or in combination with the *Arabidopsis thaliana* 1-deoxy-D-xylulose 5-phosphate synthase (*AtDXS*) gene, which supplies metabolic precursors to the carotenoid biosynthetic pathway, or with the *Arabidopsis thaliana ORANGE* (*AtOR*) gene, which promotes the formation of a metabolic sink. The authors found that the combined expression of the *ZmPSY* and the *PaCRTI* genes with the *AtDXS* gene or the *AtOR* gene determined 2.1- to 5.8-fold increase in the accumulation of carotenoids in rice endosperm compared to the expression of the *ZmPSY* and the *PaCRTI* genes alone (Table 1). In particular, significant increase was observed in the proportion of total carotenoids represented by β-carotene that was 25–39% (1.17–2.15 µg/g) in genotype expressing the *ZmPSY* and the *PaCRTI* genes alone, and 47–52% (7.50–16.61 µg/g) and 40–50% (5.87–10.52 µg/g) in genotypes expressing these genes in combination with the *AtDXS* or the *AtOR* gene, respectively [56] (Table 1). 

Alternatively, an increase in the rice endosperm carotenoid content was recently achieved by blocking the degradation carotenoids into apocarotenoids catalysed by the CCDs. In particular, Ko and coworkers [57] obtained *OsCCD*-RNAi lines that were cross-fertilized with β-carotene-producing transgenic lines; bred lines were obtained that displayed up to 1.6-fold enhancement in total carotenoids that reached values ranging from 1.43 µg/g to 3.58 µg/g (Table 1).

In addition to the development of β-carotene accumulating lines, biofortification of rice for other carotenoid molecules has been recently achieved. In particular, Ha and coworkers [91] used a combination of genetic engineering and conventional breeding to induce the accumulation, into the endosperm of japonica-type rice, of zeaxanthin, astaxanthin and capsanthin, three important carotenoids that reduce the risk of age-related macular degeneration and obesity [2,8]. To achieve this goal, β-carotene producing rice varieties were used to re-direct the carbon flux through the carotenoid pathway from β-carotene to astaxanthin and capsanthin via zeaxanthin. Three functional rice varieties were developed that contained similar levels of total carotenoids to the β-carotene producing rice varieties but higher levels of ketocarotenoids including astaxanthin (1.4 μg/g), ketoxanthophylls including capsanthin (0.4 μg/g) and zeaxanthin (0.8 μg/g). Astaxanthin biosynthesis was also engineered in Indica-type rice; biofortified rice lines were generated that accumulated up to 16.2 µg/g astaxanthin in the endosperm and showed high antioxidant activity [92].

An alternative way for the fortification of rice is represented by the ‘Vitamin and Mineral Premix Kernels’. Rice premix is made by the addition of a vitamin–mineral premix to rice flour, which is then extruded in the form of a rice kernel; alternatively, the vitamin–mineral premix is added as a coat over natural rice kernels. The first technology is preferred as it has the advantage of protecting the added micronutrients within the manufactured grains and minimizes losses during storage and cooking. A successful example of this technology is the PATH (Program for Appropriate Technology in Health)’s Ultra Rice technology that packs vitamin A in its ester form (retinyl palmitate) together with, iron, zinc, thiamine, folic acid and other B vitamins, into cold-extruded grains made from a rice flour base [93]. This rice premix is blended with rice flour in a mixing ratio of 1:100 that ensures the nutritional requirements. This blend is currently being introduced in the developing countries through collaborative efforts with the World Food Programme and it has been shown that its regular consumption can efficiently increase the overall vitamin A status of these populations.

### 5.2. Storage

Although literature is available on the factors that affect carotenoid loss during cereal storage, little information is available on the storage of ‘Golden Rice’ seeds. Very recently Bollinedi and coworkers [94] evaluated the effect of different storage conditions and storage atmosphere on the stability of β-carotene in ‘Golden Rice’. They observed that after 6 months of storage at 4 °C under air packaging β-carotene content in paddy, brown and polished ‘Golden Rice’ decreased by 68%, 72% and 79% respectively; the decrease was even higher (80%, 81% and 84%%, respectively) after storage at 25 °C. The lower β-carotene loss was observed at 25 °C when paddy rice was stored under vacuum packaging compared to air packaging (46% vs. 80%) [94]. These findings confirm that the high temperature and the presence of air in the package negatively affect β-carotene stability and highlight that also the polishing of the rice grains may contribute to accelerate the β-carotene degradation during storage; this information can be useful to set an effective storage method for ‘Golden Rice’ that safeguards its nutritional status.

A transgenic approach was also used to reduce carotenoid loss during the storage of ‘Golden Rice’ grains. Since LOX plays a role in the oxidative degradation of carotenoid pigments during seed storage, the endogenous LOX activity of high-carotenoid Indica rice seeds were silenced by RNAi technology. After artificial aging at 45 °C and 85% relative humidity for 14 days, the β-carotene loss observed in *LOX*-RNAi seeds was found to be significantly lower than that observed in the β-carotene-enriched ‘Golden Rice’ seeds (10–25% vs. 56%) [95].

Particular attention has been paid to evaluating the effect of the storage conditions on the stability of retinyl palmitate in biofortified rice premix. The retention of retinyl palmitate after storage was found to be significantly affected by the type of techniques used to make the rice premix. After 12 months of storage at mild conditions (25 ± 5 °C and 60% humidity) the greater losses of retinyl palmitate were observed in coated compared to cold- and hot-extruded premix (77% vs. 20–30%); these losses increased (93% vs. 40–50%) after 6 months of storage at higher temperature and humidity (40 ± 5 °C and 75% humidity) [96]. These findings confirm that the exposure of added micronutrients on the surface of manufactured grains favours their degradation and loss. Moreover, Lee and coworkers [97] observed that the stability of the retinyl palmitate in ‘Ultra Rice’ grains appeared to be more affected by temperature than humidity. In particular, no significant losses in the retinyl palmitate content were observed at 0 °C and 23 °C throughout 24 months of storage, whereas less than 50% of the original retinyl palmitate was retained after the same time of storage at 35 °C; no significant differences in the stability of retinyl palmitate were instead observed between 50% and 80% relative humidity throughout 24 weeks of storage at the same temperature [97]. Higher retention values were obtained by Li and coworkers [98] by using an ‘Ultra Rice’ formulation that contained butylated hydroxyanisole and butylated hydroxytoluene as the hydrophobic antioxidants and ascorbic acid as the hydrophilic antioxidant; citric acid and sodium triphosphate were also added to chelate metal ions and to stabilize moisture, respectively. This formulation retained more than 70% of the added retinyl palmitate after 24 weeks storage at 45°C. The effect of different bags on retinyl palmitate content after storage was also evaluated and it was reported that after 18 weeks of storage at 30 °C, the retention of retinyl palmitate stored in aluminium foil bags, which protect the grains from light, was 18% higher as compared to storage in polyethylene bags, which resulted in about 40% degradation [99]. 

### 5.3. Processing

Rice is used mostly at the household level, where it is mainly consumed as boiled or steamed. Unfortunately, as for the storage, the effect of cooking on the retention of β-carotene in ‘Golden Rice’ has not yet been fully elucidated. Only a preliminary work exists aimed at evaluating the effect of steam cooking on the retention of β-carotene in polished grains of different ‘Golden Rice’ genotypes. Steaming accounted for a β-carotene loss of 17–24%, with differences among genotypes that were probably due to the different amounts of water absorbed during cooking [94]. 

More information is available for biofortified rice premix. Evidence has been reported that the amount of retinyl palmitate lost by ‘Ultra Rice’ after cooking depends upon the cooking methods. In particular, a percent retention that ranged from 75% to 87% was reported, with the highest retention observed after cooking without excess water and the lowest after cooking with excess water; an intermediate (77%) retention was observed by using rice cooker in the absence of excess water [97]. Pinkaew and coworkers [99] found that, compared to ‘Ultra Rice’ that was obtained by cold extrusion, hot-extruded premix had higher retinyl palmitate retention (more than 90%) after cooking with the rice cooker. Conversely, Wieringa and coworkers [100] did not observe significant differences in the retinyl palmitate retention among the different techniques used for the production of rice premix, but, similarly to Lee and coworkers [97], reported a broad variability among different cooking methods. In particular, they observed that soaking gave a retinyl palmitate retention of 82%, whereas the retention after direct rice boiling (either in excess water or not) was only 20%; washing rice before boiling gave an intermediate retention of retinyl palmitate (54%) compared to soaking and direct boiling methods [100].

## 6. Sorghum

### 6.1. Composition

Sorghum is a diploid cereal species that is a close relative of maize. It is extensively cultivated in those extremely arid and semiarid areas where other crops such as maize cannot be grown. In particular, in Africa sorghum is a basic staple food for about 300 million people [101]. Therefore, improving the carotenoid content in sorghum might have a great impact on the health of poor people in these regions. 

Grain sorghum genotypes are designated as white- or yellow-endosperm on the basis of the carotenoid content in the endosperm [102]. According to the USDA-National Plant Germplasm System, only 381 accessions of sorghum were characterized as yellow-endosperm from a total of 42,869 accessions [103]. A recent study evaluating modern white- and yellow-endosperm lines and hybrids of sorghum showed that in fully-matured kernels the levels of total carotenoids ranged from 3.82 to 19.50 μg/g [13] (Table 1). β-Carotene, zeaxanthin and lutein were the predominant carotenoids with a content of 2.23–6.02 μg/g, 0.45–6.97 μg/g and 0.28–4.05 μg/g, respectively; a content of 0.70–3.93 μg/g was also measured for β-cryptoxanthin in most of the genotypes analysed [13] (Table 1). Considering that yellow sorghums contain provitamin A carotenoids, biofortification aimed at increasing the concentration of these compounds through plant breeding seems feasible. In this regard, a recombinant inbred line population from a yellow- by a white-endosperm parental cross was developed and QTLs associated with carotenoid content were identified [104]. In particular, five QTLs for β-carotene were mapped, with one of them that was stable across different environments, explained large proportions of the phenotypic variance and was associated with a *PSY* gene [104]. This information represents a starting point for the development of high-provitamin A sorghum lines through appropriate MAS breeding programs. Alternatively, sorghum flour has been spiked with β-carotene powder, with the guiding level of spiking that was based on the level of β-carotene in ‘Golden Rice’, which is more than 30 μg/g [105].

To improve the carotenoid synthesis in sorghum a transgenic approach has also been used. A sorghum genotype that contained low levels of carotenoids in the mature seeds, in particular low levels of all-*trans* β-carotene (0.5 µg/g), the predominant provitamin A carotenoid, was engineered with the *AtDXS*, the *ZmPSY1* and the *PaCRTI* genes; mutant lines were obtained that accumulated all-*trans* β-carotene up to 9.1 µg/g; other carotenoids, such as lutein, zeaxanthin, α-carotene, 13-*cis* β-carotene and 9-*cis* β-carotene were also increased significantly [58] (Table 1). The same authors found that the co-expression of the barley homogentisate geranylgeranyl transferase (*HGGT*) gene, which is involved in tocotrienol and tocopherol biosynthesis [106], with the genes responsible for enhancing β-carotene levels, significantly improved not only the levels of α-tocotrienol, α-tocopherol and γ-tocopherol but also the level of all-*trans* β-carotene that reached 12.3 μg/g [58]. The authors assumed that the antioxidant effect of vitamin E is important in enhancing the stability of all-*trans* β-carotene through seed maturation, potentially leading to a higher accumulation of all-*trans* β-carotene in the mature seeds. In this regard, evidence has been reported that in cereal grains a significant correlation exists between radical scavenging activity and tocol content but not with carotenoid content [107]. This finding suggests that the interplay between tocols and carotenoids in grains could be very important in carotenoid stability, since tocols could scavenge oxygen radicals more efficiently than carotenoids, thus protecting carotenoids from oxidation.

### 6.2. Storage

The effect of storage on the carotenoid content of sorghum grain has been poorly investigated. Che and coworkers [58] assessed the carotenoid stability in their biofortified transgenic sorghum lines after storage at room temperature for 4 weeks either in the dark or under constant light. They observed that in the dark the degradation of all-*trans* β-carotene was about 52% and increased to 63% under light conditions as a result of the photo-oxidative degradation [58]. As already observed for the freshly harvested seeds, the stability of the all-*trans* β-carotene in the stored grains improved by 2.6-fold when the barley *HGGT* gene was co-expressed with β-carotene biosynthetic genes [58].

### 6.3. Processing

Like the other cereals, sorghum needs to be processed before human consumption. In developing countries, sorghum grains are generally milled and used in porridge, couscous, noodles and baked products, such as unleavened bread, cookies and cakes. Cardoso and coworkers [108] evaluated the carotenoid retention in sorghum flour obtained by milling grains before and after processing with dry heat (conventional oven, microwave oven and conventional popper) and wet heat (cooking in water). The authors found that the carotenoid content remained almost unchanged (99%) after grain cooking in water, whereas a significant decrease was observed for all the treatments with dry heat. Moreover, the flours obtained by milling the grains before the processing with dry heat showed lower retention of carotenoids compared to flours obtained by milling the grains after the heat treatments (27–74% vs. 81–85%) [108]. This is probably ascribable to the increased exposure of the grain matrix to heat after milling. The same authors observed a great carotenoid loss after extrusion, with retention that ranged between 31% and 37%, probably ascribable to the sensitivity of high carotenoid compounds to temperature, pressure and shearing [109]. A slight decrease in β-carotene content that ranged between 74% and 87% was also observed when sorghum grains were soaked [110]. The retention of provitamin A was also evaluated in porridges prepared from both nontransgenic and biofortified transgenic sorghum grains. The provitamin A content ranged from 16 to 31 μg/100 g in porridges prepared with nontransgenic grains and 40−250 μg/100 g in porridges from transgenic biofortified grains; the overall provitamin A retention after cooking averaged 77% and ranged from 48% to 100% [111].

## 7. Conclusions

The quality of cereal grains is a key factor that determines the quality of the cereal-based end-products. As illustrated above, significant advances have been done in the manipulation and improvement of the carotenoid content in cereal grains for human consumption. However, storage and processing usually have a negative impact on the carotenoid content and composition of the raw and processed material. Several studies have reported an important genotypic effect on carotenoid retention during post-harvest handling of cereals. The differences among genotypes should be investigated in more detail so to provide useful information to cereal breeders to look for genetic traits linked to enhanced carotenoid stability over storage and processing. The recently emerged cheap and massive next-generation sequencing together with the new editing technologies will aid breeders to pursue this goal. Moreover, storage conditions and processing methods should be selected in order to achieve not only the maintenance of stocks and the good preparation of foods, but also preserve the health-promoting effects of the natural and fortified cereal grains in their end-products. To date the purpose of the fortification has been focused particularly on the improvement of the provitamin A carotenoids to alleviate vitamin A deficiency, which represents an important public-health issue in developing and underdeveloped countries. In the future greater attention should also be paid to the increase of non-provitamin A carotenoids, which might play a role in the prevention of chronic disease related to lifestyle habits typical of the industrialized countries.

## Figures and Tables

**Table 1:** Carotenoid content and composition in cereal grains.

Species	Fortification Strategy		Total Carotenoids (µg/g)	Lutein (µg/g)	Zeaxanthin (µg/g)	Provitamin A Carotenoids			Refs.
						β-Carotene (µg/g)	α-Carotene (µg/g)	β-Cryptoxanthin(µg/g)	
Durum wheat	Traditional breeding		1.178–4.416	0.721–3.078	0.088–0.184	0.007–0.033	0.003–0.029	0.002–0.014	[26]
	MAS		5.00–7.70	n.d.	n.d.	n.d.	n.d.	n.d.	[32]
Bread wheat	Traditional breeding		0.603–1.940	0.391–1.890	n.d.	n.d.	n.d.	n.d.	[27,28,29,30]
	Translocation lines		1.133–1.215	0.897–1.020	n.d.	n.d.	n.d.	n.d.	[30]
	Transgenesis	*ZmPSY+PaCRTI*	2.31–4.96	n.d.	n.d.	n.d.	n.d.	n.d.	[37]
		*PaCRTB+PaCRTI*	3.21–4.76	0.29–0.36	0.38–0.53	2.14–3.21	0.19–0.34	0.18–0.29	[38]
		*HYD-RNAi+PaCRTB*	8.28–9.31	1.65–1.97	0.42–0.69	3.98–5.04	0.22–0.37	0.12–0.25	[39]
Maize	Traditional breeding		9.55–62.96	1.23–23.93	1.44–32.40	0.31–3.27	0.45–2.65	0.13–5.17	[44,45]
	MAS		n.d.	3.3–25.7	0.7–44.7	1.6–26.0	n.d.	3.6–13.0	[46]
			n.d.	n.d.	n.d.	1.9–21.7	n.d.	n.d.	[47]
	Transgenesis	*PaCRTB+PaCRTI*	16–47	2.93–6.68	3.41–6.21	2.98–8.64	0.28–4.65	0.26-0.74	[48]
		*RNAi-ZmBCH2*	68–106	7–23	13–19	21–30	n.d.	3–11	[49]
		*ZmPSY+PaCRTI*	up to 156.14	up to 9.76	up to 25.36	up to 57.35	up to 6.10	up to 5.97	[50]
Rice	Traditional breeding		n.d.	0.036–0.109	0.014–0.037	0.066-0.150	n.d.	n.d.	[14]
	Transgenesis	*NpPSY+PaCRTI (Golden Rice)*	up to 1.6	n.d.	n.d.	up to 0.7	n.d.	n.d.	[51,52,53]
		*NpPSY+PaCRTI (Golden Rice-1)*	up to 6.0	n.d.	n.d.	n.d.	n.d.	n.d.	[54]
		*ZmPSY+PaCRTI (Golden Rice 2)*	36.7	n.d.	n.d.	30.9	4.8	0.4	[55]
		*ZmPSY+PaCRTI*	4.61–5.51	0.16–0.26	0.03–0.17	1.17–2.15	0.70–1.90	0.00–0.17	[56]
		*ZmPSY+PaCRTI+AtDXS*	17.79–31.78	0.26–0.69	0.13–0.33	7.50–16.61	3.60–9.70	0.39–0.41	[56]
		*ZmPSY+PaCRTI+AtOR*	11.53–25.83	0.55–1.73	0.23–0.57	5.87–10.52	3.20–9.70	0.19–1.13	[56]
		*OsCCD-RNAi*	1.43–3.58	0.22–0.58	0.06–0.11	0.76–2.04	0.14–0.61	0.16–0.32	[57]
Sorghum	Traditional breeding		3.82–19.50	0.28–4.05	0.45–6.97	2.23–6.02	n.d.	0.70–3.93	[13]
	Transgenesis	*ZmPSY+PaCRTI+AtDXS*	n.d.	6.94–12.01	3.61–5.43	2.5–9.1	n.d.	n.d.	[58]
		*ZmPSY+PaCRTI+AtDXS+HGGT*	n.d.	9.32–11.46	4.04–5.95	7.3–12.3	n.d.	n.d.	[58]
n.d.: not determined									
